# Socioeconomic status and parent perceptions about the costs and benefits of youth sport

**DOI:** 10.1371/journal.pone.0258885

**Published:** 2021-11-10

**Authors:** Emily Kroshus, Pingping Qu, Sara Chrisman, Stanley Herring, Frederick Rivara

**Affiliations:** 1 Seattle Children’s Research Institute, Center for Child Health, Behavior and Development, Seattle, WA, United States of America; 2 Department of Pediatrics, University of Washington, Seattle, WA, United States of America; 3 Harborview Injury Prevention and Research Center, Seattle, WA, United States of America; 4 Division of Adolescent Medicine, University of Washington, Seattle, WA, United States of America; 5 Department of Rehabilitation Medicine, University of Washington, Seattle, WA, United States of America; University of Jyvaskyla, FINLAND

## Abstract

**Objectives:**

Describe what costs and benefits parents across the socioeconomic spectrum weight most heavily when making decisions about sport participation for their children.

**Method:**

Cross-sectional survey of a nationally representative online panel of parents of children between the ages of 5 and 18 (n = 1025, 52% response rate). Parents rated the importance of a series of potential costs and benefits of youth sport and these responses were compared across tertiles of per capita family income. We first examined the association between family income tertiles and cost and benefit variables. Model-based cluster analysis was then used to identity homogeneous groups of responses to costs and benefits.

**Results:**

In all income tertiles, the top two benefits of sport were the same: having fun and being physically active. Sport as a means of keeping children out of trouble was very important for 64% of low-income parents as compared to 40% of high-income parents. Obtaining a college athletic scholarship was very important for 26% of low-income parents, as compared to 8% of high-income parents. Relative rankings of potential costs were similar by income tertile, with risk of concussion and other injury and the impact of sport on schoolwork prioritized across tertiles.

**Conclusions:**

Parents prioritized fun and fitness in sport, and were concerned about injury and the impact of sport on academics. Lower income parents were the most likely to view keeping their child out of trouble, and the potential for a college athletics scholarship, as benefits of sport. Efforts to support parental decision making should be grounded in an understanding that family preferences are contextually constrained. While all parents should be appropriately informed about the potential costs and benefits they are weighting in their sports-related decision making, such family-focused efforts should be balanced with the recognition that structural change is needed to address income-related concerns about sport participation.

## Introduction

Sport can be an important way for youth to meet recommended levels of physical activity [1, 2], and has the potential to play an important role in psychosocial development [3]. Parents often play a central role in deciding whether and when their child will initiate organized sports participation and what sports they will play [4]. Shortly after starting organized sport, many families have to make additional decisions about sport participation, including whether and when their child will progress to an (often expensive and time consuming) competitive or “travel” team, and whether their child will “specialize” or focus only on one sport [5, 6]. Such parental decision making can be viewed through a relative risk framework [7], where costs (monetary and non-monetary) and benefits are subjective and context dependent. Understanding how parents prioritize different costs and benefits of youth sport can guide approaches to supporting informed and context-relevant decision-making that allows for sustained youth sport participation.

One key determinant of parental perceptions of the costs and benefits of sport may be their socioeconomic status—their relative and absolute economic, educational and occupational resources that shape health inequalities [8]. We focus specifically on the economic dimension of this construct given its centrality to socioeconomic status, high degree of correlation between income, education and occupation [8], and the theorized link between monetary resources and sport selection [9, 10]. Across all ages, organized sport participation tends to be lowest in low socioeconomic status families [11, 12]. Among children in higher income households (income of $100,000/year or more), only 12% did not participate in any sport during the past year, whereas among children from lower income households (income of less than $25,000/year) 30% did not participate in sport [11]. Youth sport participation typically requires expenditures of time and money, which pose a relatively larger burden on less affluent families [13, 14]. Less affluent families may struggle providing transport to practices and games [13, 15] due to less flexible work schedules and having less access to a means of transportation. The reinforcing value of possible future benefits of sport participation, like securing an athletic scholarship, may also vary by a family’s socioeconomic status [16].

The family’s physical and social environment may also contribute to how costs and benefits of youth sport are prioritized. Income-based residential stratification is prevalent in the United States [17], heightened among families with children [18] and among Black as compared to non-Hispanic white households due to structural racism [17]. In communities characterized by low income residents, there tend to be fewer options for sport participation as compared to higher resource communities [13] and the built environment is often less conducive to outdoor exercise or free-play [19, 20]. For example, in three US states (North Carolina, New York, and Maryland), the lowest income tertile of communities were found to be 4.5 times less likely to have public recreational facilities such as sports fields and pools are compared to the highest income tertile of communities [21]. Thus, the ability of parents in these settings to be selective in sport choice may be limited, potentially leading to lower importance placed on any given (positive or negative) attribute of a possible sport for their child. On the other hand, more socioeconomically disadvantaged parents tend to have elevated concern about health issues more broadly [22], potentially due to their perceived lack of agency in limiting negative outcomes [23].

The goal of the present paper was to learn more about how families across the income spectrum prioritize different potential costs (both financial and non-financial) and benefits of youth sport. We sought to compare absolute responses between income groups and the relative ranking of costs and benefits within income groups. We did not make directional hypotheses, but anticipated that there would be differences in both the absolute and relative rankings by income group. Second, we explored how individual parent and child characteristics were related to the patterning of parent prioritization of costs and benefits of organized youth sport.

## Methods

### Sample and procedure

This study was approved by the University of Washington and Seattle Children’s Research Institute’s Institutional Review Boards. Waiver of documentation of informed consent was obtained because data were not identifiable. We conducted a cross-sectional online survey of 1025 parents, using a nationally representative panel (response rate = 52%). Data collection was facilitated by the market research company GfK (www.gfk.com), with a sampling frame of their 60,000 person online panel. This panel is designed to reflect the composition of 97% of the US population, with probability-based sampling of the Delivery Sequence File of the US Postal Service used to identify potential panel members, who are then provided Internet and computer hardware if needed to participate. Data collection occurred in October 2017. Individuals aged 18 and older residing in the US were eligible for inclusion if they were the parent of at least one child between 5 and 18 years of age. Current or prior child sport participation was not a criterion for participation. The questionnaire was available in both English and Spanish. When the responding parent had more than one child between the ages of 5 and 18, they were instructed to answer with reference to the child whose birthday was next. Feedback on questionnaire wording and content was obtained through cognitive interviews with convenience sample of six parents of youth athletes between the ages of 5 and 17. Questions were then pilot tested with 62 parents meeting survey eligibility criteria described above to ensure response variability. Additional detail on the development and pilot testing process has been described elsewhere [22].

### Measures

#### Potential costs and benefits of sport participation

Parents indicated how important eight potential benefits would be in their decision to allow their child to play a given organized sport: be physically active, develop teamwork skills, keep out of trouble, improve strength and stamina, obtain a college athletic scholarship, make friends, learn how to be a good winner and loser, and have fun. Four response options were provided: very important, somewhat important, not very important and not at all important. Parents indicated how important seven potential costs would be in their decision to allow their reference child to play a given organized sport: time for other activities, cost of sport participation, how to get child to and from practices and games, risk of concussion, risk of injury (other than concussion), emotional stress of sport, impact on schoolwork/homework. Four response options were provided: very much a concern, somewhat of a concern, not very much of a concern, not at all a concern. Items were generated based on extant qualitative literature about potential costs and benefits of youth sport participation [6, 24–26], and wording was refined through cognitive interviews (n = 6) with members of our target population.

#### Per-capita family income

Annual family income was queried in six categories, the lowest being under $25,000 and the highest being $250,000 and over. Per-capita family income was calculated by dividing the mid-point of the income category by household size, referencing the US Census per capita poverty threshold guidelines [27]. For the >$250,000 category, a mid-point of $402,000 was used [28]. Per-capita family income was divided into three categories (low, moderate and high) using a tertile split.

#### Race/Ethnicity

Parent race and ethnicity were queried using aggregated categorizations from the US Census (2010): white, non-Hispanic; black, non-Hispanic; other, non-Hispanic; Hispanic.

#### Parent education

Parents reported their highest level of formal education completed, which was subsequently grouped into three categories: high school diploma or less, some college, bachelor’s degree or higher.

#### Urbanicity

Using the March 2017 Current Population Survey categories (Metro, Non-Metro), households were classified by whether or not they are located in a metropolitan statistical area.

#### Contact sport viewership

Parents indicated whether during the past 12 months they had watched or attended in person the following: NFL football, college football, ice hockey, boxing, mixed marital arts, pro wrestling, major league soccer, or international professional soccer, NBA basketball, college basketball. Responses were dichotomized into any viewership (1) and no viewership (0).

#### Other demographic characteristics

Parent and child age and gender identity (male, female) were reported.

### Analysis

We first examined the association between family income tertiles and the four-level cost and benefit variables using Pearson chi-square tests or Fisher’s exact tests (where appropriate for small cell sizes). Next, we rank ordered costs and benefits within each income tertile by the percentage of respondents who indicated that this factor was either very important (benefits) or very much a concern (costs). Model based cluster analysis was then used to identity homogeneous groups of responses to costs and benefits, using these dichotomized response variables. Listwise deletion was used for four cases that were missing most responses to costs and benefits, leaving a sample size of 1021. To determine how many clusters there were in the population, we ran the cluster analysis with varying number of clusters from 1 to 8, choosing the optimal number by examining the Bayesian Information Criterion (BIC), where smaller BIC indicated a better fit. After determining the best fitting number of clusters, we calculated the probability that parents within that cluster would think a benefit was very important or cost very much a concern. We next examined associations between the cluster memberships and all other variables in both univariate and multivariate logistic regression analyses. Analyses were conducted in R version 3.4.4, with the model-based cluster analysis (latent profile analysis) using the R poLCA package. An alpha value of 0.05 was used as the threshold for statistical significance.

## Results

Female parents comprised 56% of the sample, mean parent age was 42.57 (SD = 8.67) and mean reference child age was 7.61 (SD = 4.01). Slightly more than half of respondents were non-Hispanic white. Mean per family capita income for parents in the lowest income tertile was $7,699, $21,161 for the middle tertile, and $53, 212 for the highest tertile. Additional sample characteristics are reported in Table 1.

**Table 1:** Sample characteristics.

Continuous variables		Mean	SD
Parent age (n = 1025)		42.57	8.67
Reference child age (n = 1014)		7.61	4.01
**Categorical Variables**		n	%
Parent respondent gender	Female	569	56.23
	Male	456	43.77
Reference child gender	Female	497	49.03
	Male	522	50.97
Parent race/ethnicity	White, Non-Hispanic	657	56.57
	Black, Non-Hispanic	98	11.37
	Hispanic	199	22.62
	Others, Non-Hispanic	71	9.44
Parent education	Bachelor’s degree or higher	393	36.41
	Less than high school/high school	352	36.31
	Some college	280	27.28
Per-capita family income	Low	344	34.33
	Middle	380	35.55
	High	301	31.12
Contact sport viewership	No	706	77.98
	Yes	204	22.02
Urbanicity	Metro	901	87.08
	Non-Metro	124	12.92

For two of the listed benefits there were significant differences in importance by income (Table 2). Keeping out of trouble was very important for 64% of low-income parents as compared to 48% of middle-income parents and 40% of high-income parents (p<0.001). Obtaining a college athletic scholarship was very important for 26% of low-income parents, as compared to 13% of middle-income and 8% of high-income parents (p<0.001). Across all income tertiles, the top two potential benefits of sport by rank order were the same: having fun and being physically active (Table 3).

**Table 2:** Comparison of cost and benefit considerations in sport participation by income tertiles.

		Low income		Middle income		High income		
Variable	Level of importance^1^ or concern^2^	n	%	n	%	n	%	p
**Benefits**								
Be physically active (n = 1023)	1	245	71%	263	69%	219	73%	0.742
	2	82	24%	105	28%	73	24%	
	3	10	3%	8	2%	6	2%	
	4	6	2%	3	1%	3	1%	
Develop teamwork skills	1	234	68%	257	68%	199	66%	0.801
	2	98	29%	107	28%	90	30%	
(n = 1021)	3	7	2%	6	2%	8	3%	
	4	3	1%	8	2%	4	1%	
Keep out of trouble	1	219	64%	182	48%	120	40%	<0.001
(n = 1022)	2	80	23%	114	30%	92	31%	
	3	29	8%	48	13%	67	22%	
	4	14	4%	35	9%	22	7%	
Improve strength and stamina	1	193	57%	179	47%	140	47%	0.066
	2	115	34%	162	43%	136	45%	
(n = 1019)	3	26	8%	32	8%	21	7%	
	4	7	2%	5	1%	3	1%	
Obtain a college athletic scholarship	1	87	26%	51	13%	25	8%	<0.001
	2	102	30%	75	20%	45	15%	
(n = 1020)	3	95	28%	153	40%	134	45%	
	4	56	16%	100	26%	97	32%	
Make friends	1	189	55%	204	54%	160	53%	0.964
(n = 1021)	2	128	37%	139	37%	115	38%	
	3	20	6%	29	8%	21	7%	
	4	5	1%	7	2%	4	1%	
Learn how to be a good winner/loser	1	236	69%	235	62%	185	61%	0.286
	2	90	26%	123	32%	94	31%	
	3	11	3%	13	3%	17	6%	
(n = 1023)	4	6	2%	8	2%	5	2%	
Have fun	1	274	80%	296	79%	247	82%	0.950
(n = 1021)	2	63	18%	73	19%	48	16%	
	3	4	1%	4	1%	3	1%	
	4	3	1%	4	1%	2	1%	
**Cost**								
Time for other activities	1	99	29%	81	22%	58	19%	0.015
	2	164	48%	177	47%	154	51%	
(n = 1019)	3	60	17%	92	24%	76	25%	
	4	20	6%	26	7%	12	4%	
Cost of sport participation	1	141	41%	104	28%	47	16%	<0.001
	2	148	43%	159	42%	112	37%	
(n = 1018)	3	41	12%	82	22%	107	36%	
	4	11	3%	33	9%	33	11%	
Getting child to practices/games	1	106	31%	80	21%	53	18%	<0.001
	2	128	37%	123	33%	110	37%	
(n = 1020)	3	70	20%	119	31%	101	34%	
	4	38	11%	56	15%	36	12%	
Risk of concussion	1	190	55%	143	38%	123	41%	<0.001
(n = 1019)	2	99	29%	133	35%	104	35%	
	3	40	12%	86	23%	59	20%	
	4	14	4%	15	4%	13	4%	
Risk of injury (other than concussion)	1	179	52%	123	33%	108	36%	<0.001
	2	108	31%	167	44%	109	36%	
(n = 1021)	3	48	14%	77	20%	71	24%	
	4	8	2%	11	3%	12	4%	
Emotional stress of sport	1	108	31%	85	22%	48	16%	<0.001
	2	146	43%	144	38%	122	41%	
(n = 1021)	3	64	19%	120	32%	105	35%	
	4	25	7%	29	8%	25	8%	
Impact on schoolwork	1	174	51%	146	39%	114	38%	0.011
(n = 1021)	2	118	34%	155	41%	125	42%	
	3	41	12%	59	16%	52	17%	
	4	10	3%	18	5%	9	3%	

^1^Importance levels: 1 = very important, 2 = somewhat important, 3 = not very important, 4 = not at all important.

^2^Concern levels: 1 = very much a concern, 2 = somewhat of a concern, 3 = not very much of a concern, 4 = not at all a concern.

**Table 3:** Rank ordering of parent perceptions of the most important benefits and costs of youth sport by income tertile.

	Low income		Middle income		High income	
Ranking		%		%		%
**Benefit**						
1	Have fun	80%	Have fun	78%	Have fun	82%
2	Be physically active	71%	Be physically active	69%	Be physically active	73%
3	Learn to be a good winner and loser	69%	Develop teamwork skills	68%	Develop teamwork skills	66%
4	Develop teamwork skills	68%	Learn to be a good winner and loser	62%	Learn to be a good winner and loser	62%
5	Keep out of trouble	64%	Make friends	54%	Make friends	53%
6	Improve strength and stamina	57%	Keep out of trouble	48%	Improve strength and stamina	47%
7	Make friends	55%	Improve strength and stamina	47%	Keep out of trouble	40%
8	Obtain a college athletic scholarship	26%	Obtain a college athletic scholarship	14%	Obtain a college athletic scholarship	8%
**Cost**						
1	Risk of concussion	55%	Impact on schoolwork	39%	Risk of concussion	41%
2	Risk of injury (non-concussion)	52%	Risk of concussion	38%	Impact on schoolwork	38%
3	Impact on schoolwork	51%	Risk of injury (non-concussion)	32%	Risk of injury (non-concussion)	36%
4	Cost of sport participation	41%	Cost of sport participation	28%	Time for other activities	19%
5	Emotional stress of sport	32%	Emotional stress of sport	22%	Getting child to practices/games	18%
6	Getting child to practices/games	31%	Time for other activities	22%	Emotional stress of sport	16%
7	Time for other activities	29%	Getting child to practices/games	21%	Cost of sport participation	16%

Note: Percentage listed is the percentage of parents in that income terile who indicated that each cost or benefit was “very much a concern” or “very important” in sport choice decisions.

For each potential cost, a larger percentage of low income parents indicated that it was very much a concern than did middle and high-income parents (Table 2). Rankings of costs were similar by tertile (Table 3). Among the lowest income parents, the top three costs were risk of concussion (55%), risk of other injury (52%) and impact on schoolwork/homework (51%). Among the highest income families, the top concerns were risk of concussion (41%), impact on schoolwork (38%) and risk of injury other than concussion (36%). Emotional stress from sport participation was ranked fifth highest for low and middle income families, and 6^th^ for high income families.

Results of the cluster analysis indicated that a model with 4 clusters was the best fit for the data (see S1 Data for BIC values by number of clusters). The probability that parents in each cluster thought that each benefit or cost was very important or very much a concern is reported in Table 4. Cluster 1 (41% of the sample) is characterized by heightened focus on all benefits except obtaining a college athletic scholarship (“Focus on Benefits”). Cluster 2 (23% of the sample) is characterized by heightened focus on both costs and benefits (“Costs and Benefits”). Cluster 3 (18% of the sample) is characterized by heightened concern about concussion, injury other than concussion and the impact of sport on academics (“Injury and Academics”). Cluster 4 (18% of the sample) is characterized by a relatively greater proportion of parents focused on fun and being physically active as compared to other potential benefits (“Fun and Fitness”); we note that the absolute proportion of parents in this cluster endorsing either of these attributes is lower than the other clusters.

**Table 4:** Probability that parents within each cluster think each benefit or cost is very important or very much a concern when thinking about sports selection for their child.

Variable	Cluster 1	Cluster 2	Cluster 3	Cluster 4
	*Focus on Benefits*	*Costs and benefits*	*Injury and academics*	*Fun and fitness*
**Benefit**				
Be physically active	0.92	0.94	0.36	0.29
Develop teamwork skills	0.92	0.98	0.23	0.18
Keep out of trouble	0.57	0.92	0.23	0.12
Improve strength and stamina	0.66	0.84	0.09	0.11
Obtain a college athletic scholarship	0.14	0.40	0.05	0.02
Make friends	0.74	0.82	0.15	0.13
Learn to be a good winner and loser	0.86	0.94	0.23	0.17
Have fun	0.95	0.97	0.56	0.49
**Cost**				
Time for other activities	0.08	0.54	0.38	0.04
Cost of sport participation	0.17	0.55	0.42	0.07
Transportation	0.12	0.50	0.37	0.01
Risk of concussion	0.21	0.92	0.79	0.05
Risk of injury (other than concussion)	0.14	0.91	0.72	0.04
Emotional stress of sport	0.05	0.62	0.41	0.00
Impact on schoolwork	0.24	0.83	0.66	0.09

Proportion of sample by cluster: Cluster 1 = 0.41, Cluster 2 = 0.23, Cluster 3 = 0.18, Cluster 4 = 0.18.

Univariate comparisons of the probability of cluster membership found significant differences by income category. High-income (49%) and middle-income (44%) parents were most likely to be in Cluster 1 (*Focus on benefits*), whereas low-income parents (34%) were most likely be in Cluster 2(*Costs and benefits*). Univariate comparisons of the probability of cluster membership by other measured demographic are presented in Table 5. Multiple logistic regression with stepwise variable selection (Tables 6–8) was used to separately compare the likelihood of a parent being in Clusters 1 (*Focus on benefits*), 2 (*Costs and benefits*), and 3 (*Injuries and academics*) versus Cluster 4 (*Fun and fitness*). Parents were more likely to be in Cluster 1 (*Focus on benefits*) rather than Cluster 4 (*Fun and fitness*) when they were viewers of contact sports (OR = 1.8, 95% CI = 1.15–2.82) and when they were younger (OR = 0.98, 95% CI = 0.96–1.00). Parents were more likely to be in Cluster 2 (*Costs and benefits*) rather than Cluster 4 (*Fun and fitness*) when they were younger (OR = 0.96, 95% CI = 0.93–0.98) and Black non-Hispanic (OR = 2.53, 95% CI = 1.2–5.35) or Hispanic (OR = 8.25, 95% CI = 3.81–17.86) as compared to White non-Hispanic, and in the middle income category (OR = 0.50, 95% CI = 0.28–0.89, where the lowest income tertile is the reference group). Finally, parents were more likely to be in Cluster 3 (*Injuries and academics*) as compared to Cluster 4 (*Fun and fitness*) when they were female (OR = 1.61, 95% CI = 1.02–2.54), Hispanic (OR = 3.65, 95% CI = 1.66–8.04), and in the middle income category (OR = 0.56, 95% CI = 0.32–0.97, where the lowest income tertile is the reference group). We note that demographic characteristics including metro/non-metro region and parent education were not significantly associated with cluster membership in multivariate analyses. Parent contact sport viewership was associated with greater likelihood of being in Cluster 1 (*Focus on benefits*) than Cluster 4 (*Fun and fitness)* (OR = 1.80, 95% CI = 1.15–2.82, p = 0.011).

**Table 5:** Univariate associations of cluster membership with all other variables.

Variable		Cluster 1	Cluster 2	Cluster 3	Cluster 4	p
		*Focus on benefits*	*Costs and benefits*	*Injuries and academics*	*Fun and fitness*	
**Continuous variables** ^**1**^						
Parent age		43.05 (8.49)	40.86 (9)	42.42 (8.66)	43.93 (8.3)	0.002
Child age		7.64 (3.94)	6.95 (3.9)	8.01 (4.29)	8.04 (3.94)	0.018
**Categorical variables** ^**2**^						
Parent gender	Male	203 (45%)	90 (20%)	66 (15%)	95 (21%)	0.006
	Female	224 (40%)	140 (25%)	114 (20%)	89 (16%)	
Child gender	Male	221 (42%)	118 (23%)	89 (17%)	93 (18%)	0.945
	Female	202 (41%)	111 (22%)	90 (18%)	91 (18%)	
Parent education	Less than high school/high school	128 (36%)	116 (33%)	60 (17%)	47 (13%)	<0.001
	Some college	113 (41%)	57 (21%)	52 (19%)	56 (20%)	
	Bachelor’s degree or higher	186 (47%)	57 (15%)	68 (17%)	81 (21%)	
Parent race/ethnicity	White, Non-Hispanic	303 (46%)	98 (15%)	109 (17%)	145 (22%)	<0.001
	Black, Non-Hispanic	31 (32%)	34 (35%)	19 (19%)	14 (14%)	
	Others, Non-Hispanic	27 (38%)	16 (23%)	16 (23%)	12 (17%)	
	Hispanic	66 (34%)	82 (42%)	36 (18%)	13 (7%)	
Income	Low	112 (33%)	115 (34%)	70 (20%)	46 (13%)	<0.001
	Middle	168 (44%)	66 (17%)	62 (16%)	82 (22%)	
	High	147 (49%)	49 (16%)	48 (16%)	56 (19%)	
Contact sport viewership	No	269 (38%)	158 (22%)	137 (19%)	139 (20%)	0.001
	Yes	110 (54%)	37 (18%)	24 (12%)	33 (16%)	
Urbanicity	Non-Metro	46 (37%)	28 (23%)	21 (17%)	29 (23%)	0.384
	Metro	381 (42%)	202 (23%)	159 (18%)	155 (17%)	

^1^Mean (SD).

^2^N (%).

**Table 6:** Stepwise multivariate logistic regression of parent being in cluster 1 *(Focus on benefits*) vs. cluster 4 (*Fun and fitness*) (N = 529; R-square = 0.03).

Variable	Level	Odds Ratio	LL	UL	P
Parent age		0.98	0.96	1	0.044
Parent contact sport viewership	No	(ref)	–	–	–
	Yes	1.80	1.15	2.82	0.011
Urbanicity	Non-Metro	(ref)	–	–	–
	Metro	1.60	0.94	2.75	0.086

**Table 7:** Stepwise multivariate logistic regression of parent being in cluster 2 (*Costs and benefits*) vs. cluster 4 (*Fun and fitness*) (N = 343; R-square = 0.21).

Variable	Level	Odds Ratio	LL	UL	P
Parent age		0.96	0.93	0.98	0.002
Parent race/ethnicity	White, Non-Hispanic	(ref)	–	–	–
	Black, Non-Hispanic	2.53	1.2	5.35	0.015
	Others, Non-Hispanic	1.31	0.53	3.19	0.559
	Hispanic	8.25	3.81	17.86	0
Income	Low	(ref)	–	–	–
	Middle	0.50	0.28	0.89	0.018
	High	0.87	0.46	1.62	0.657

**Table 8:** Stepwise multiple logistic regression of parent being in cluster 3 (*Injuries and academics)* vs. cluster 4 (*Fun and fitness*) (N = 321; R-square = 0.08).

Variable	Level	Odds Ratio	LL	UL	P
Parent gender	Male	(ref)	–	–	–
	Female	1.61	1.02	2.54	0.042
Parent race/ethnicity	White, Non-Hispanic	(ref)	–	–	–
	Black, Non-Hispanic	1.43	0.64	3.16	0.38
	Others, Non-Hispanic	1.65	0.73	3.74	0.229
	Hispanic	3.65	1.66	8.04	0.001
Income	Low	(ref)	–	–	–
	Middle	0.56	0.32	0.97	0.039
	High	0.7	0.39	1.29	0.253

## Discussion

In this national sample, parents across the income spectrum were similar in terms of what they saw as the most important benefits of sport (fun and physical activity) and what they saw as the most concerning potential costs (injury and impact on schoolwork). Beyond these priorities, there were notable differences by income category in the relative importance parents assigned to keeping their child out of trouble and obtaining a college scholarship (relatively more important for lower income families), the monetary cost of sports participation (relatively more important for lower income families) and time for other activities (relatively more important for more affluent families). Across all potential costs, absolute concern was greatest among the lowest income parents and high-income families were more likely to be in the two clusters focused on benefits—Cluster 1 (*Focus on Benefits*) and Cluster 4 (*Fun and Fitness*).

The finding that parents tended to prioritize fun and physical activity is consistent with developmentally appropriate models of youth sport participation [3, 29]. “Fun” in a youth sport context has been operationalized as a multi-dimensional construct that includes a social/relational dimension (i.e., team friendships), positive coaching, and process orientation (i.e., learning and improving) [30]. We note that child age did not explain differences in the types of factors parents prioritized (i.e., cluster membership). This suggests that the types of considerations parents report prioritizing in sport choice are largely unrelated to their child’s developmental stage. Parents who watched more contact sports on television were less likely to be in the cluster focused on fun and fitness than in the cluster focused on benefits. Further research is needed to explore the pathways through which such viewership relates to youth sport priorities (i.e., is viewership an indicator of sport participation history, or determinant of attitudes towards sport achievement). High viewership parents may be an appropriate target for interventions that emphasize the importance of fun and developmentally appropriate youth sport participation.

The fact that across all families, around one in ten considered the potential of a college athletic scholarship to be a very important consideration for sport choice may reflect universal misappraisal of underlying probabilities. This is consistent with prior research among parents of youth baseball players that found that more than one-third believed their child was likely to secure a college athletic scholarship [31]. Fewer than 10% of high school athletes participate in their sport in college, and only 2% (i.e., 0.2% of high school athletes) obtain an athletic scholarship covering some fraction of their expenses [32]. For some families, this perceived potential benefit of a given sport (i.e., obtaining a college scholarship) might offset other sport risks (e.g., risk of concussion) when making decisions about sport participation. It is critical that parents are adequately informed about the very low probability of a college athletic scholarship so that this can be appropriately weighted in sport choice decision making. As the youth sports industrial complex continues to grow, this information may not reliably come from individuals with a vested financial stake in a given child’s sport participation. Thus, different channels for knowledge translation, such as from primary care physicians, should be explored.

Across all potential costs, absolute concern was greatest among the lowest income parents. Conversely, high-income families were more likely to be in the two clusters focused on benefits—Cluster 1 (*Focus on Benefits*) and Cluster 4 (*Fun and Fitness*), whereas lower income families were more likely to be in Cluster 2 (*Costs and Benefits*). Some of the differences in perceived costs (e.g., cost of sport, transport to games and practices) may be a direct function of lower income families having fewer financial resources, less flexible work schedules and less access to a car [33]. Differences in absolute levels of concern by income may also reflect a perceived capacity by higher income families to mitigate potential costs as a result of their health literacy (e.g., knowledge and ability to take appropriate steps to address injury concerns) and community assets (e.g., access to healthcare to help reduce injury-related harm). Further research is needed to understand the reasons for these observed between-income group differences in perceived costs to identify whether there are feasible opportunities for intervention to in an effort to decrease inequities in organized sport participation.

We note that monetary cost of sport participation was ranked similarly (4^th^) in both the lowest and middle-income tertiles, while it was the lowest ranked concern in the highest income tertile. Organized youth sport may be placing a financial burden on a large fraction of US families, including the middle class [10]. This would be consistent with evidence that middle class families are engaging in intentional and resource intensive child rearing practices [34], both as a result of social pressure and a desire to give their child every advantage at a time of widening income inequality and stagnating wages for the lower and middle class. Critically, it is not clear that resource and time intensive youth sports are more beneficial for youth than lower pressure and less burdensome options; conversely, it is possible they put youth at risk of burnout and sport attrition [35]. Ensuring that all families are able to make an informed choice about sport participation, rationally appraising the costs and benefits of different options (e.g., expensive travel league vs. in-town recreational league) is important for helping families play sports in a way that works for their child and is in balance with other family resource demands.

### Implications for research and practice

The present findings indicate that sport choice considerations vary across families. Given this heterogeneity and subjectivity, helping families make informed decisions about sport choice may best be facilitated by a shared decision-making approach [36]. Shared decision-making presumes that there is no one “correct” decision, and aims to help families make decisions that are informed and consistent with what matters most to them. Consistent with such an approach, accurate and easily understood information should be shared with parents about the likelihood that their child will experience a range of potential positive and negative sports-related outcomes (e.g., injury, athletic scholarship). Support or messaging could potentially also be provided to help families reflect critically on their values related to youth sport participation. More research is needed to understand how to translate information about variability in parental sport choice considerations into usable interventions to support developmentally appropriate youth sport participation, and the settings in which this information should be shared and discussed. However, such family-centered recommendations do not address broader structural barriers to family decision making about sport participation, such as relatively less access to recreational facilities in low income communities [21]. Efforts to equitably increase youth sport participation should also attend to structural barriers—a more difficult proposition than supporting informed choice that may require collective action for policy change and resource allocation within communities and between communities.

### Limitations

These findings are specific to the United States, may not generalize to other cultures or settings. The survey response rate means that the sample may not be nationally representative. Although we measured metro/non-metro location and found that it was not related to cluster membership, we did not assess whether location interacted with income to explain sport choice priorities. Future research in larger samples that allow for fully powered sub-group analyses should further explore how the present results generalize across settings and subgroups of families. Individuals identifying as Asian, Native Hawaiian or Pacific Islander, American Indian or Alaska Native were grouped into a single category of “Other, non-Hispanic.” This group includes individuals with vastly different cultural practices and experiences of discrimination that may impact their parenting in relation to sport. Further research in larger samples is needed to better understand whether there are differences in sport parenting among less prevalent racial and ethnic groups. Responses may overweight the perspectives of parents of younger children as the mean child age was 7.6 years. Survey questions did not assess actual youth sport participation, parent sport participation or health conditions that may influence their perspectives on youth sport. There may be interactions between child age and other child characteristics such as their current sport involvement and preferences and parent characteristics such as their own sport history that are unmeasured in the present study and that influence sport preference construction. Further, questions were only with reference to one child (with the next birthday)—however the broader family environment, including number of children, their ages, and their current and prior participation in sport may have been influential in family preference construction. Finally, sport choice may be driven by emotional influences (e.g., sense of belonging or cultural affinity for a given sport), making deliberative parent beliefs about costs and benefits a relatively small influence on a family’s decision. The present study addresses how parents construct preferences about sport choice; further research is needed to understand how these articulated preferences relate to actual decision making related to sport choice.

## Conclusion

Parents in this sample prioritized fun and fitness in sport, and were concerned about injury and the impact of sport on academics. Lower income parents were the most likely to view keeping their child out of trouble, and the potential for a college athletics scholarship, as benefits of sport. Overall, lower income parents were more likely to focus on balancing costs and benefits in their sport choice decisions, whereas higher income parents were more focused on benefits. Efforts to support parental decision-making should be grounded in an understanding that family preferences are contextually constrained. While all parents should be appropriately informed about the potential costs and benefits they are weighting in their sports-related decision making, such family-focused efforts should be balanced with the recognition that structural change is needed to address income-related concerns about sport participation.

## Supporting information

S1 Data (XLSX)

## References

[1] World Health Organization. Physical activity and young people. World Health Organization. Published 2018. Accessed January 28, 2019. https://www.who.int/dietphysicalactivity/factsheet_young_people/en/

[2] Kann L. Youth Risk Behavior Surveillance—United States, 2017. *MMWR Surveill Summ*. 2018;67. doi: 10.15585/mmwr.ss6708a1 29902162 PMC6002027

[3] Youth sport programs: an avenue to foster positive youth development: Physical Education and Sport Pedagogy: Vol 10, No 1. Accessed March 3, 2020. https://www.tandfonline.com/doi/abs/10.1080/1740898042000334890

[4] Wiersma LD, Fifer AM. “The Schedule Has Been Tough But We Think It’s Worth It”: The Joys, Challenges, and Recommendations of Youth Sport Parents. *J Leis Res*. 2008;40(4):505–530. doi: 10.1080/00222216.2008.11950150

[5] Shaw SM, Dawson D. Purposive Leisure: Examining Parental Discourses on Family Activities. *Leis Sci*. 2001;23(4):217–231. doi: 10.1080/01490400152809098

[6] Chard C, Edwards J, Potwarka L. Understanding the Perceived Attributes and Consequences of Participation in Youth “Rep” Hockey. *J Appl Sport Manag*. 2015;7(2):10.

[7] Weber EU, Blais A-R, Betz NE. A domain-specific risk-attitude scale: Measuring risk perceptions and risk behaviors. *J Behav Decis Mak*. 2002;15(4):263–290.

[8] Wolfe J. The Effects of Socioeconomic Status on Child and Adolescent Physical Health: An Organization and Systematic Comparison of Measures. *Soc Indic Res*. 2015;23(1). Accessed March 3, 2020. https://link.springer.com/article/10.1007/s11205-014-0733-4

[9] Andersen PL, Bakken A. Social class differences in youths’ participation in organized sports: What are the mechanisms? *Int Rev Sociol Sport*. 2019;54(8):921–937. doi: 10.1177/1012690218764626

[10] Post EG, Green NE, Schaefer DA, et al. Socioeconomic status of parents with children participating on youth club sport teams. *Phys Ther Sport*. 2018;32:126–132. doi: 10.1016/j.ptsp.2018.05.014 29793120

[11] Katzmarzyk PT, Denstel KD, Beals K, et al. Results from the United States 2018 Report Card on Physical Activity for Children and Youth. *J Phys Act Health*. 2018;15(s2):S422–S424. doi: 10.1123/jpah.2018-0476 30475143

[12] McMillan R, McIsaac M, Janssen I. Family Structure as a Correlate of Organized Sport Participation among Youth. *PLoS ONE*. 2016;11(2). doi: 10.1371/journal.pone.0147403 26863108 PMC4749258

[13] Race Mohan L. and its impact on youth sport participation choice. *Int J Sport Stud*. 2014;4(11):1309–1316.

[14] Theokas C, Bloch M. Out-of-School Time Is Critical for Children: Who Participates in Programs? Research-to-Results Fact Sheet. *Publication #2006–20*. *Child Trends*; 2006.

[15] Beets MW, Cardinal BJ, Alderman BL. Parental Social Support and the Physical Activity-Related Behaviors of Youth: A Review. *Health Educ Behav*. 2010;37(5):621–644. doi: 10.1177/1090198110363884 20729347

[16] Singer JN. Benefits and Detriments of African American Male Athletes’ Participation in a Big-Time College Football Program. *Int Rev Sociol Sport.* 2008;43(4):399–408. doi: 10.1177/1012690208099874

[17] Reardon SF, Bischoff K. Income Inequality and Income Segregation. *Am J Sociol*. 2011;116(4):1092–1153. doi: 10.1086/657114 21648248

[18] Owens A. Inequality in Children’s Contexts: Income Segregation of Households with and without Children. *Am Sociol Rev*. 2016;81(3):549–574.

[19] Gordon-Larsen P, Nelson M, Page P, Popkin BM. Inequality in the Built Environment Underlies Key Health Disparities in Physical Activity and Obesity. *Pediatrics*. 117(2). Accessed March 3, 2020. https://pediatrics.aappublications.org/content/117/2/417.short doi: 10.1542/peds.2005-0058 16452361

[20] Estabrooks PA, Lee RE, Gyurcsik NC. Resources for physical activity participation: Does availability and accessibility differ by neighborhood socioeconomic status? *Ann Behav Med*. 2003;25(2):100–104. doi: 10.1207/S15324796ABM2502_05 12704011

[21] Moore LV, Diez Roux AV, Evenson KR, McGinn AP, Brines SJ. Availability of Recreational Resources in Minority and Low Socioeconomic Status Areas. *Am J Prev Med*. 2008;34(1):16–22. doi: 10.1016/j.amepre.2007.09.021 18083446 PMC2254179

[22] Kroshus E, Qu P, Chrisman SPD, Schwien C, Herring SA, Rivara FP. Parental concern about concussion risk for their children. *Soc Sci Med*. 2019;222:359–366. doi: 10.1016/j.socscimed.2018.12.037 30674437

[23] Matthews KA, Gallo LC, Taylor SE. Are psychosocial factors mediators of socioeconomic status and health connections? A progress report and blueprint for the future. *Ann N Y Acad Sci*. 2010;1186:146–173. doi: 10.1111/j.1749-6632.2009.05332.x 20201872

[24] Neely KC, Holt NL. Parents’ Perspectives on the Benefits of Sport Participation for Young Children. *Sport Psychol.* 2014;28(3):255–268. doi: 10.1123/tsp.2013-0094

[25] Holt NL, Kingsley BC, Tink LN, Scherer J. Benefits and challenges associated with sport participation by children and parents from low-income families. *Psychol Sport Exerc*. 2011;12(5):490–499. doi: 10.1016/j.psychsport.2011.05.007

[26] Merkel DL. Youth sport: positive and negative impact on young athletes. *Open Access J Sports Med*. 2013;4:151–160. doi: 10.2147/OAJSM.S33556 24379720 PMC3871410

[27] US Census. Household Income: HINC-06. The United States Census Bureau. Accessed March 3, 2020. https://www.census.gov/data/tables/time-series/demo/income-poverty/cps-hinc/hinc-06.html

[28] Data. United States Census Bureau. Accessed March 3, 2020. https://www.census.gov/programs-surveys/cps/data.html

[29] Bergeron MF, Mountjoy M, Armstrong N, et al. International Olympic Committee consensus statement on youth athletic development. *Br J Sports Med*. 2015;49(13):843–851. doi: 10.1136/bjsports-2015-094962 26084524

[30] Visek AJ, Achrati SM, Mannix HM, McDonnell K, Harris BS, DiPietro L. The Fun Integration Theory: Toward Sustaining Children and Adolescents Sport Participation. *J Phys Act Health*. 2015;12(3):424–433. doi: 10.1123/jpah.2013-0180 24770788 PMC4201634

[31] Post EG, Rosenthal MD, Rauh MJ. Attitudes and Beliefs towards Sport Specialization, College Scholarships, and Financial Investment among High School Baseball Parents. *Sports*. 2019;7(12):247. doi: 10.3390/sports7120247 31835455 PMC6955882

[32] Probability of Competing Beyond High School. NCAA. Published December 17, 2013. Accessed March 3, 2020. http://www.ncaa.org/about/resources/research/probability-competing-beyond-high-school

[33] Humbert ML, Chad KE, Spink KS, et al. Factors That Influence Physical Activity Participation Among High- and Low-SES Youth. *Qual Health Res*. 2006;16(4):467–483. doi: 10.1177/1049732305286051 16513991

[34] Smyth C, Craig L. Conforming to intensive parenting ideals: willingness, reluctance and social context. *Fam Relatsh Soc*. 6(1):107–124.

[35] Dunn CR, Dorsch TE, King MQ, Rothlisberger KJ. The Impact of Family Financial Investment on Perceived Parent Pressure and Child Enjoyment and Commitment in Organized Youth Sport. *Fam Relat*. 2016;65(2):287–299. doi: 10.1111/fare.12193

[36] Opel DJ. A 4-Step Framework for Shared Decision-making in Pediatrics. *Pediatrics*. 2018;142(Supplement 3):S149–S156. doi: 10.1542/peds.2018-0516E 30385621

